# Association between RoPE score and PFO grading on bubble echocardiography in cryptogenic stroke patients: a retrospective cohort study

**DOI:** 10.3389/fstro.2025.1676220

**Published:** 2026-01-09

**Authors:** Saeedur Rahman, Erik Hendrickson, Jamie Henderson, Samuel McGrath, Ayah Mekhaimar, Kishen Mathi, Jake Hudson, Robert Sargent, Brian Clapp

**Affiliations:** 1 Department of Stroke Medicine, Darent Valley Hospital, Dartford, United Kingdom; 2 University of California, San Diego, La Jolla, CA, United States; 3 Department of Cardiology, Guy's and St Thomas' Hospital, London, United Kingdom; 4 Department of Cardiology, Darent Valley Hospital, Dartford, United Kingdom

**Keywords:** cryptogenic stroke, paradoxical embolization, patent foramen ovale, RoPE score, clinical prediction of high risk PFO features

## Abstract

**Introduction:**

Identification of high-risk anatomical and physiological features of a patent foramen ovale (PFO) is important for patient selection for transcatheter device closure of PFO in patients with cryptogenic stroke. Currently, there are no clinical screening tools in use that can be used in predicting high-risk PFO features before undertaking transoesophageal echocardiography.

**Methods:**

This retrospective cohort study, conducted in a stroke unit in South East England, included 130 patients diagnosed with ischaemic stroke or transient ischaemic attack who were deemed as cryptogenic in nature following initial evaluation (≤55 years with no known risk factors or immediately identified underlying etiology). Patients underwent comprehensive diagnostic evaluations, including bubble echocardiography. The primary predictor, risk of paradoxical embolism (RoPE) score (≥6), was assessed for its association with a significant PFO, categorized as model 1 (≥small) and model 2 (≥moderate). Multivariable logistic regression models were used to estimate adjusted odds ratios for the relationship between RoPE score and PFO presence.

**Results:**

Of the 130 patients, 47 had a known etiology, and 83 had cryptogenic stroke. The known etiology group had higher rates of hypertension, hyperlipidaemia, and non-stenotic atherosclerosis, while the cryptogenic group had more cortical strokes and higher RoPE scores. Multivariable analysis showed that a lower RoPE score (≤5) was associated with known etiology (aOR: 3.91, *p* < 0.01). RoPE scores ≥6 were significantly associated with both small and moderate PFOs (aORs: 5.39, *p* < 0.01 and 15.95, *p* < 0.01, respectively). Of 28 candidates for PFO closure, 20 underwent the procedure, all with high RoPE scores and large PFOs.

**Discussion:**

This study reinforces the importance of a multidisciplinary approach in the evaluation and management of patients with PFO and suspected embolic stroke. While PFO is prevalent in both cryptogenic and non-cryptogenic stroke patients, its pathogenic role is highly context dependent. Our findings confirm that a high RoPE score (≥6) and a cortical stroke phenotype are independently associated with clinically relevant, higher-grade PFOs. Furthermore, patients selected for device closure consistently exhibited high RoPE scores and multiple high-risk anatomical features, aligning with current international guidelines. Importantly, low RoPE scores (≤5) were significantly associated with strokes of known etiology, underscoring the utility of the RoPE score not only in identifying likely PFO-related strokes but also in ruling out embolic mechanisms. These results support the integration of clinical scoring systems like RoPE for patient selection about the suitability for device closures as higher RoPE scores predict high-risk PFO and therefore minimize unnecessary interventions.

**Conclusion:**

RoPE scores may be utilized in predicting high-risk anatomical and physiological features of PFO. However, larger prospective studies are needed to validate these findings and refine pre-transoesophageal echocardiography screening tools.

## Introduction

Stroke is one of the most dreaded cardiovascular diseases. According to a statement released by the World Stroke Organization on World Stroke Day 2024, stroke is a leading cause of mortality and morbidity worldwide. Each year, approximately 12 million people worldwide experience their first episode of stroke. The most alarming statistics is that one in four adults above the age of 25 will experience a cerebrovascular event in their lifetime. Traditionally, stroke is thought to be a disease of the elderly population. However, 16% of all strokes happen in individuals below the age of 50 (World Stroke Organization, 2025).

The majority of strokes (approximately 87%) are ischaemic in nature (American Stroke Association, 2021). According to the American Stroke Association guidelines on the evaluation and management of cryptogenic stroke, of all the ischaemic strokes, 35% to 45% are cryptogenic in nature. The definition of “cryptogenic stroke” differs based on the classification used [TOAST classification; causative classification of stroke (CCS)] (Arsava et al., 2010; Adams et al., 1993). However, a simplified definition of cryptogenic stroke is when an underlying etiology has not been identified after initial investigation including routine blood test, brain imaging, vascular imaging, prolonged cardiac monitoring, and transthoracic echocardiogram. An ischaemic stroke is also thought to be cryptogenic if there are two or more competing etiologies.

Success of secondary prevention measures is dependent on identifying the correct underlying pathophysiological process. This was demonstrated by landmark trials like NAVIGATE ESUS (Hart et al., 2018) and RE-SPECT ESUS (Diener et al., 2019) involving embolic stroke of undetermined source (ESUS), which showed that blind direct oral anticoagulant anticoagulation does not offer benefits over standard treatment by aspirin. This brought the definition of ESUS into question, and subsequent trials have highlighted underestimated risk of etiologies like aortic arch atheroma (Sakai et al., 2022) and non-stenotic carotid disease (Tao et al., 2021). This highlights the importance of treating the correct underlying reason to prevent recurrent events.

Patent foramen ovale (PFO) has long been identified as a potential underlying etiology in cryptogenic stroke. PFO is a conduit for paradoxical embolism leading to stroke. However, PFO is common among the general population (25%) and a causal relationship between PFO and the index event needs careful consideration. This is more important than ever before as device closure of PFO has emerged as the most effective evidence-based treatment for this group of patients. Studies have shown that PFO closure in patients with competing possible etiologies has higher recurrence rates compared to patients where all other possible causes have been ruled out (Mariucci et al., 2017). It also raises the question of avoidable procedure related complications and may increase inappropriate healthcare expenditures.

The risk of paradoxical embolism (RoPE) score is a well-validated tool for estimating “PFO attributable fraction” at an individual level in cryptogenic stroke, with higher scores indicating a greater probability of strokes related to PFO (Kent et al., 2013). This is used in conjunction with the PFO-Associated Stroke Causal Likelihood (PASCAL) classification, which allows stroke physicians, vascular neurologists, and cardiologists to make evidence-based decisions regarding PFO closure. PASCAL classification depends on high-risk PFO features, some of which can only be determined through transoesophageal echocardiogram such as tunnel length and height (Kent et al., 2021). High-risk PFO features are a key element in decision-making regarding PFO closure, especially the PFO shunt grade. Multiple studies have externally validated RoPE scores in predicting the association between PFO and cryptogenic stroke (Strambo et al., 2021). Although the Spencer scale can be used to quantify a shunt using transcranial Doppler bubble study, no studies have investigated the association with PFO grading assessed by bubble echocardiogram (Park et al., 2021).

We conducted a retrospective observational study in patients who were labeled as cryptogenic stroke to assess the strength of the association between a moderate to high RoPE score and higher PFO grades on bubble echocardiogram in a stroke unit in the United Kingdom.

## Methodology

### Study design and population

This retrospective, observational cohort study was conducted in the stroke unit at a large district general hospital in South East England, covering the period from 1 January 2022 to 31 August 2024. We screened adult patients (aged ≥18 years) who were ≤ 55 years of age who were admitted to the stroke unit or evaluated in the transient ischaemic attack (TIA) outpatient clinic during this period (148). Patients who later were identified as stroke mimics were excluded from the final analysis. One hundred and thirty (130) patients were deemed eligible to be included in the study who had clinically or radiologically confirmed stroke or TIA and were deemed cryptogenic in nature. Cryptogenic stroke was defined as patients under the age of 55, absence of vascular stenosis or atrial fibrillation, and not known to have hypertension, diabetes, or hyperlipidaemia at the point of initial diagnosis. They subsequently underwent comprehensive evaluation including routine screening for vascular risk factors, neuro and vascular imaging, thrombophilia screening, 14-day cardiac monitoring, and a bubble echocardiogram.

### Data collection and outcomes measures

Baseline demographic data were collected including age (as a continuous variable), sex, and ethnicity. Clinical data such as risk factors categorized by obesity (BMI > 30), smoking status, alcohol misuse, history of hypertension, history of diabetes, hyperlipidaemia, history of stroke or TIA, ischaemic heart disease, peripheral vascular disease, vascular stenosis/dissection, atrial fibrillation, thrombophilia, antiphospholipid syndrome, hyper viscosity syndrome, and polycythemia were also included. Data were collected on stroke classification based on imaging findings (cortical vs. lacunar infarcts). The primary exposure variable was a moderate to high RoPE score (≥6), while the main outcome of interest was the presence of a significant PFO with differing thresholds (model 1: ≥small; model 2: ≥moderate). PFO grading was based on the number of bubbles seen within the first three cardiac cycles in the left atrium (grade 0 = no shunt, grade 1 = small shunt/1–5 bubbles, grade 2 = moderate shunt/6–19 bubbles, grade 3 = large shunt/>20 bubbles, grade 4 = very large/complete opacification) (Akagi, 2021).

### Statistical analysis

Descriptive statistics were calculated for baseline demographic and clinical characteristics. Continuous variables were presented as medians with interquartile ranges (IQR), while categorical variables were summarized as frequencies and percentages. Group comparisons for categorical variables were performed using Fisher exact tests; the Wilcoxon rank-sum test was utilized for continuous variables given the non-normal distribution variables.

To assess the association between the RoPE score and the presence of a known etiology or a cryptogenic classification, a multivariable logistic regression model was employed. This model estimated the adjusted odds ratios (aOR) for having a low RoPE score and a known etiology relative to a cryptogenic classification, with appropriate adjustment for potential confounders. A second set of multivariable logistic regression models were used to evaluate the adjusted odds of having a moderate to high RoPE score in relation to different thresholds for significant PFO. Covariates that demonstrated statistical significance at the *p* < 0.10 level in univariate analyses were included in the multivariable models to adjust for potential confounding. Variables directly included in the composite RoPE score (e.g., age, history of hypertension) were excluded from the adjusted model to mitigate multicollinearity and ensure the integrity of the estimates.

Receiver operating characteristics (ROC) curves were used to evaluate the diagnostic performance of the models. For each model, a ROC curve was generated, and the area under the curve (AUC) was calculated to assess the model's ability to discriminate between positive and negative cases.

Statistical significance was defined as *p* < 0.05. All analyses were performed using R (version 4.4.1).

## Results

**Figure 1:**
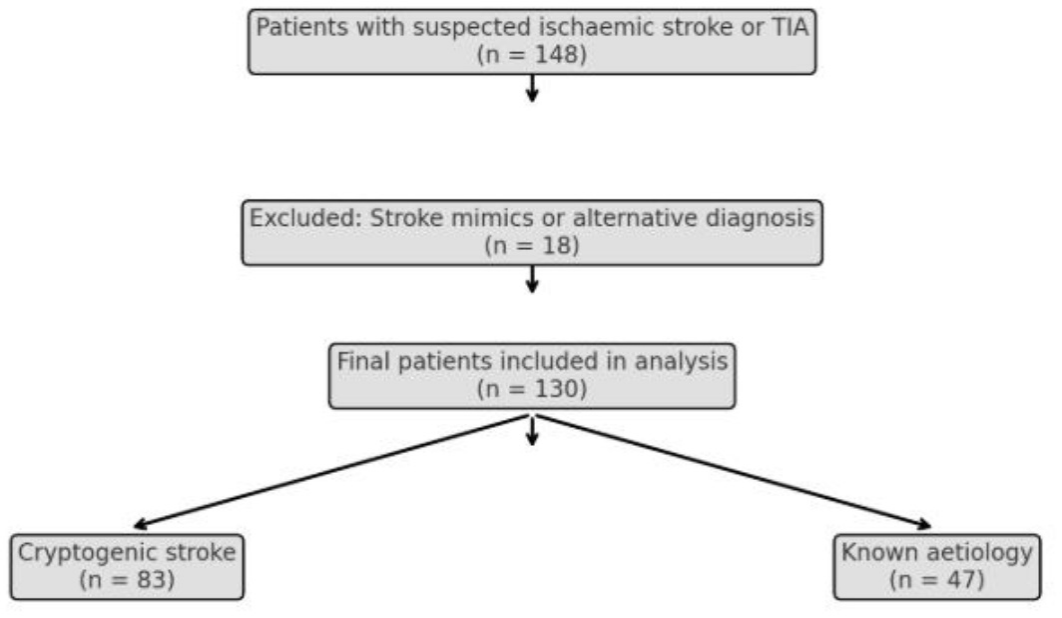
Flow diagram of participant selection.

A total of 148 patients were initially identified. Subsequently, 18 patients were rejected due to non-stroke diagnosis giving a total number of 130 stroke patients. Forty-seven patients were identified to have an underlying etiology during the course of their evaluation, and 83 were truly cryptogenic (Figure 1).

Among patients who were later found to have an underlying cause for their stroke (*n* = 47), the majority were white (64.0%), male (65.9%), and had a median age of 48 (IQR 40–56). Most were overweight (median BMI 27.1, IQR 23.5–30.5). A significant proportion of patients were current smokers (61.7%) and had a history of alcohol misuse (29.8%). Additionally, a high proportion of patients were hypertensive and had hyperlipidaemia (72.3% and 55.3%, respectively). Significant vascular stenosis and antiphospholipid syndrome were two other major underlying etiologies (27.6% and 17%, respectively). In this cohort of patients, the majority had lacunar strokes or TIA with at least two known cardiovascular risk factors. Of these patients, 55% (*n* = 26) had a positive bubble study. The presence of a significant PFO was evaluated at two distinct thresholds: the first defined significance equal to or greater than small PFO (51.1%), while the second defined significance equal to or greater than moderate PFO (34.0%) (see Table 1).

**Table 1:** Characteristics of the study population.

	**Known etiology (*****N =*** **47)**		**Crytogenic (*****N =*** **83)**		***p*-value**
	***n***	**%**	***n***	**%**	
**Overall demographics**					
Median Age (IQR)	48 (40–56)		52 (38.5–65.5)		0.202
Sex (Male)	31	65.9%	51	61.5%	0.7061
Ethnicity (White)	30	64.0%	52	62.7%	1.000
**Risk factors**					
Median BMI (IQR)	27.1 (23.5–30.5)		27 (24.5–29.5)		0.1486
Smoker	29	61.7%	35	42.1%	0.0443
Alcohol misuse	14	29.8%	15	18.1%	0.1316
History of Hypertension	34	72.3%	10	12.0%	< 0.0001
History of Diabetes	7	15.0%	6	7.2%	0.2234
Hyperlipidemia	26	55.3%	27	32.5%	0.0155
History of Myocardial Infarction	2	4.2%	1	< 1%	0.2961
History of stroke of TIA	4	8.5%	5	6.0%	0.722
Peripheral vascular disease	1	< 1%	0	0.0%	0.3615
Non-stenotic atherosclerosis	11	23.4%	7	8.4%	0.0319
Cortical Stroke	19	40.0%	58	69.9%	0.0015
Significant stenosis/Dissection	13	27.7%	0	0.0%	< 0.0001
Atrial Fibrillation	3	6.0%	0	0.0%	0.0453
APLS	8	17.0%	0	0.0%	0.0002
PFO positive	26	55.0%	39	47.0%	0.4655
Risk of Paradoxical Embolism (RoPE) score (>5 pts)	20	43.0%	66	79.5%	< 0.0001
**Outcome**					
Presence of significant PFO Model 1 (≥Small)	24	51.1%	45	54.2%	0.8550
Presence of significant PFO model 2 (≥Moderate)	16	34.0%	33	39.8%	0.5750

TIA, transient ischaemic attack; APLS, antiphospholipid antibody syndrome; PFO, patent foramen ovale; RoPE, risk of paradoxical embolism; IQR, interquartile ratio.

Among cryptogenic patients (*n* = 83), most were white (62.7%), male (61.5%), and had a median age of 52 years (IQR 38.5–65.5). Most were overweight or obese (median BMI 35, IQR 24.5–29.5) with a large proportion as current smokers (42.1%), who misuse alcohol (18.1%), and suffer from hyperlipidaemia (32.5%), hypertension (12.0%), and diabetes (7.2%). Few had a history of a stroke (6.0%) and non-stenotic atherosclerosis (8.4%), but many had evidence of a cortical stroke (69.9%). Additionally, most cryptogenic stroke patients had a RoPE score greater than or equal to six (79.5%). The presence of a significant PFO was evaluated at two distinct thresholds, the first defined significance equal to or greater than small PFO (54.2%), while the second defined significance equal to or greater than moderate PFO (40.0%).

Significant differences were observed between the two population subgroups based on various demographic and clinical characteristics. While age, sex, and ethnicity were not significantly different, notable differences were found in several risk factors. Smoking status was significantly higher in the known etiology group compared to the cryptogenic group. A history of hypertension, hyperlipidaemia, and non-stenotic atherosclerosis was considerably more prevalent in the known etiology group. In contrast, the cryptogenic group had significantly higher proportions of cortical strokes and RoPE scores greater than five. There were no significant differences in the presence of PFO between the two groups, highlighting the high prevalence of PFO in this population. These findings highlight the distinct risk profiles and underlying conditions between the two groups, with those in the known etiology group exhibiting more cardiovascular risk factors, while the cryptogenic group demonstrated a higher prevalence of cortical stroke and stronger association with elevated RoPE scores.

### RoPE score and strokes with known etiology

Univariable analysis demonstrated that current smoking status (OR: 2.21, 95% CI: 1.06–4.59, *p* = 0.03), history of hypertension (OR: 19.09, 95% CI: 7.61–47.88, *p* < 0.01), hyperlipidaemia (OR: 2.57, 95% CI: 1.23–5.36, *p* = 0.01), and non-stenotic atherosclerosis (OR: 3.32, 95% CI: 1.19–9.27, *p* = 0.02) were significantly associated with and had greater odds of having a known etiology compared to those without these individual factors (Table 2). Inversely, the presence of a cortical stroke was significantly associated with and less likely to have a known etiology compared to those without a cortical stroke presence (OR: 0.29, 95% CI: 0.14–0.62, *p* < 0.01). The RoPE score equal to or less than five was also significantly associated with and more likely to have a known etiology compared to those with a RoPE score greater than five (OR: 5.34, 95%CI: 2.39–11.51, *p* < 0.01). In multivariable models adjusting for alcohol, BMI greater than 30, hyperlipidaemia, and non-stenotic atherosclerosis as covariates, the association between a RoPE score equal to or less than five and a known etiology remained robust (aOR: 3.91, 95%CI: 1.68–9.09, *p* < 0.01, AIC: 156.31).

**Table 2:** Logistic regression of known etiology.

	**Univariable**		**Multivariable model 1 (AIC 156.31)**	
	**OR (95% CI)**	***P*** **-value**	**OR (95% CI)**	***P*** **-value**
**Overall demographics**				
Age (< 55 years)	2.46 (0.92–6.60)	0.07		
Sex (male)	1.22 (0.58–2.57)	0.61		
Ethnicity (white)	1.05 (0.50–2.21)	0.89		
**Risk factors**				
BMI (>30)	2.33 (0.96–5.66)	0.06	1.61 (0.60–4.32)	0.34
Smoker	2.21 (1.06–4.59)	0.03		
Alcohol misuse	1.92 (0.83–4.45)	0.13	1.32 (0.51–3.42)	0.57
History of hypertension	19.09 (7.61–47.88)	< 0.01		
History of diabetes	2.25 (0.71–7.13)	0.17		
Hyperlipidaemia	2.57 (1.23–5.36)	0.01	2.17 (0.97–4.85)	0.06
History of stroke of TIA	1.45 (0.37–5.69)	0.59		
Non stenotic atherosclerosis	3.32 (1.19–9.27)	0.02	1.76 (0.56–5.50)	0.33
Cortical Stroke	0.29 (0.14–0.62)	< 0.01		
Risk of Paradoxical Embolism (RoPE) score (< 6pts)	5.24 (2.39–11.51)	< 0.01	3.91 (1.68–9.09)	< 0.01

TIA, transient ischaemic attack; RoPE, risk of paradoxical embolism; OR, odds ratio.

**Figure 2:**
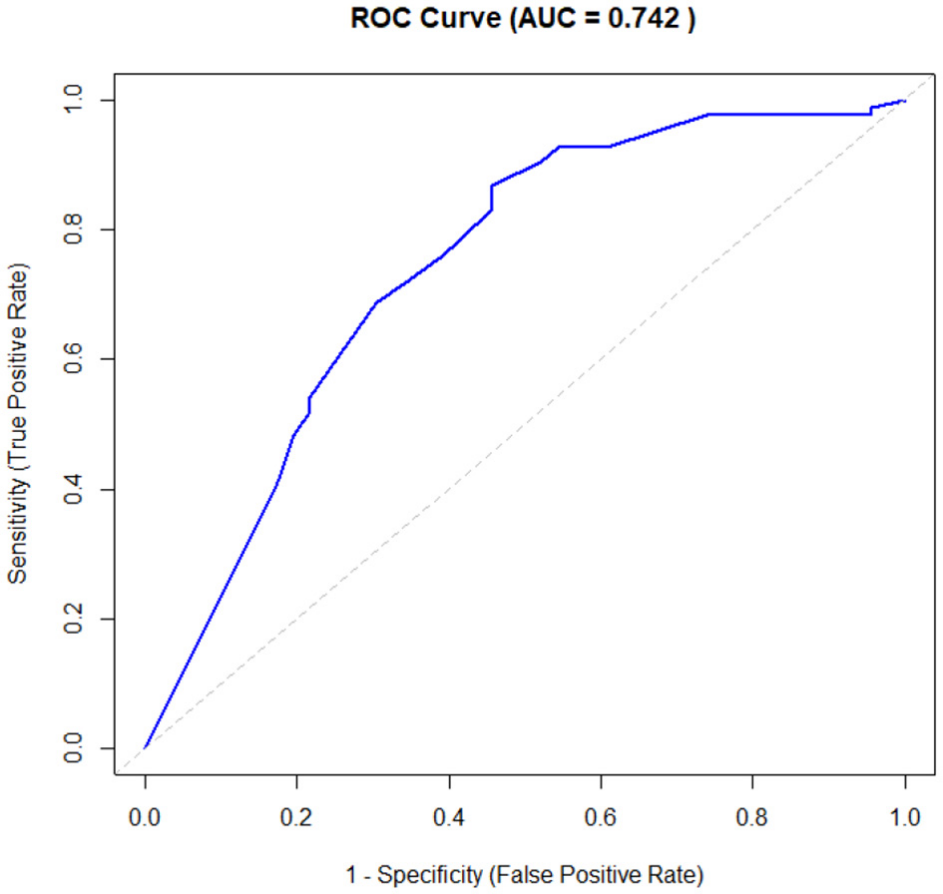
Predictive value of RoPE score to differentiate between cryptogenic stroke and stroke with known etiology. RoPE, risk of paradoxical embolism; AUC, area under the curve.

The performance of the multivariable logistic regression model for distinguishing between patients with known etiology and those with cryptogenic stroke was assessed using ROC curve analysis, yielding an AUC of 0.742 (see Figure 2). This indicates that the model, which included RoPE score as the primary predictor and adjusted for alcohol misuse, BMI ≥ 30, hyperlipidaemia, and non-stenotic atherosclerosis, demonstrates moderate discriminatory power. The AUC value suggests that the model can effectively differentiate between the two subgroups.

### RoPE score and significant PFO: model 1

Univariable analysis demonstrated that current smoking status (OR: 0.37, 95% CI: 0.15–0.90, *p* = 0.03), alcohol misuse (OR: 0.24, 95% CI: 0.07–0.83, *p* = 0.02), and a history of hypertension (OR: 0.17, 95% CI: 0.03–0.88, *p* = 0.03) were significantly associated with and less likely to have a significant PFO greater or equal to a “small PFO” compared to those without these individual factors (Table 3). Inversely, the presence of a cortical stroke was significantly associated with and more likely to have a significant PFO greater or equal to a “small PFO” compared to those without a cortical stroke presence (OR: 4.89, 95% CI: 1.75–13.65, *p* < 0.01). A RoPE score equal to or greater than six was also significantly associated with and more likely to have a significant PFO equal to or greater to a “small PFO” compared to those without a RoPE score equal to or less than five (OR: 5.33 95% CI: 1.56–18.16, *p* < 0.01). In multivariable models adjusting for alcohol as a covariate, the association between a RoPE score equal to or greater than six and a significant PFO equal to or greater than a “small PFO” remained robust (aOR: 5.39, 95% CI: 1.53–18.95, *p* < 0.01, AIC:106.82).

**Table 3:** Logistic regression of significant PFO model 1 (≥small PFO).

	**Univariable**		**Multivariable model 1 (AIC 106.82)**	
	**OR (95% CI)**	***p*** **-value**	**OR (95% CI)**	***p*** **-value**
**Overall demographics**				
Age (< 55 years)	0.76 (0.28–2.05)	0.59		
Sex (male)	1.32 (0.54–3.20)	0.54		
Ethnicity (white)	1.18 (0.48–2.88)	0.71		
**Risk factors**				
BMI (>30)	0.82 (0.24–2.79)	0.75		
Smoker	0.37 (0.15–0.90)	0.03		
Alcohol misuse	0.24 (0.07–0.83)	0.02	0.24 (0.06–0.86)	0.03
History of hypertension	0.17 (0.03–0.88)	0.03		
History of diabetes	0.40 (0.07–2.29)	0.30		
Hyperlipidaemia	0.87 (0.35–2.18)	0.76		
History of stroke of TIA	0.00 (0.00–Inf)	^*^		
Non-stenotic atherosclerosis	0.31 (0.06–1.68)	0.17		
Cortical stroke	4.89 (1.75–13.65)	< 0.01		
RoPE score (>5 pts)	5.33 (1.56–18.16)	< 0.01	5.39 (1.53–18.95)	< 0.01

TIA, transient ischaemic attack; RoPE, risk of paradoxical embolism; OR, odds ratio.

### RoPE score and significant PFO: model 2

Univariable analysis demonstrated that individuals with a history of alcohol misuse were significantly less likely to have a significant PFO (≥ moderate shunt), compared to those without a history of alcohol misuse (OR: 0.08, 95% CI: 0.01–0.65, *p* = 0.02; Table 4). In contrast, the presence of a cortical stroke was significantly associated with and more likely to have a significant PFO greater or equal to a “moderate PFO” compared to those without a cortical stroke presence (OR: 13.20, 95% CI: 2.85–61.24, *p* < 0.01). A RoPE score equal to or greater than six was also significantly associated with and more likely to have a PFO equal to or greater than a “moderate PFO” compared to those without a RoPE score equal to or less than five (OR: 15.06, 95% CI: 1.89–120.20, *p* = 0.01). In multivariable models adjusting for alcohol as a covariate, the association between a RoPE score equal to or greater than six and a significant PFO equal to or greater than a “moderate PFO” remained robust (aOR: 15.95, 95% CI: 1.95–130.45, *p* < 0.01, AIC:95.16).

**Table 4:** Logistic Regression of significant PFO (≥Moderate PFO).

	**Univariable**		**Multivariable model 2 (AIC 95.16)**	
	**OR (95% CI)**	***P*** **-value**	**OR (95% CI)**	***p*** **-value**
**Overall demographics**				
Age (< 55 years)	0.56 (0.21–1.51)	0.26		
Sex (male)	2.27 (0.88–5.85)	0.09		
Ethnicity (white)	1.33 (0.53–3.34)	0.54		
**Risk factors**				
BMI (>30)	0.72 (0.20–2.63)	0.62		
Smoker	0.43 (0.17–1.10)	0.08		
Alcohol misuse	0.08 (0.01–0.65)	0.02	0.08 (0.01–0.62)	0.02
History of hypertension	0.14 (0.02–1.18)	0.07		
History of diabetes	0.28 (0.03–2.52)	0.26		
Hyperlipidaemia	0.84 (0.33–2.17)	0.73		
History of stroke of TIA	0.00 (0.00–inf)	^*^		
Non-stenotic atherosclerosis	0.58 (0.11–3.19)	0.53		
Cortical stroke	13.20 (2.85–61.24)	< 0.01		
**Risk of paradoxical embolism (RoPE) score (>5 pts)**	15.06 (1.89–120.20)	0.01	15.95 (1.95–130.45)	< 0.01

TIA, transient ischaemic attack; RoPE, risk of paradoxical embolism; OR, odds ratio.

**Figure 3:**
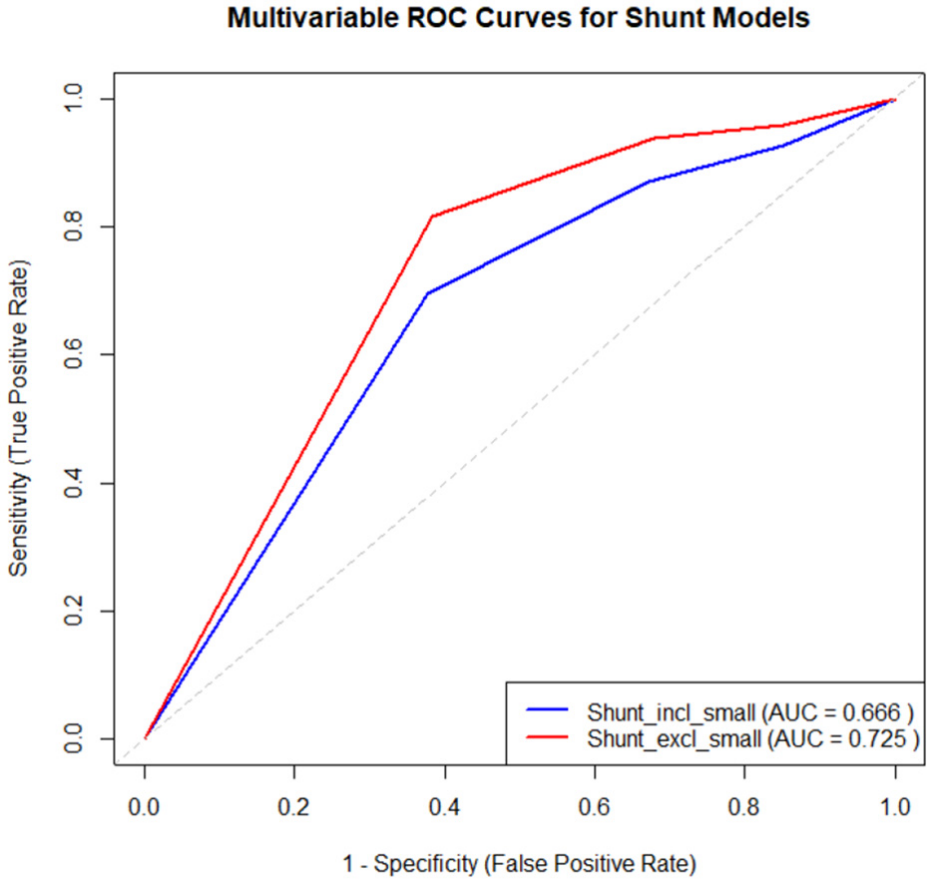
Performance of RoPE score in predicting significant PFO (model 1: ≥small PFO; model 2: ≥moderate PFO). RoPE, risk of paradoxical embolism.

The performance of the two multivariable logistic regression models for predicting shunt size (including small vs. excluding small as the PFO cutoff) was evaluated using ROC curves (Figure 3). For the model with a significant PFO greater or equal to a “small PFO” as the dependent variable, the model yielded an AUC of 0.666, indicating a moderate level of discriminatory power. In contrast, the model with a significant PFO greater or equal to a “moderate PFO” as the dependent variable demonstrated a higher AUC of 0.725, suggesting better performance in distinguishing between significant and non-significant shunts when small shunts are excluded. Both models used RoPE scores greater than five as the main predictor, with alcohol use as the sole adjustment covariate. These results suggest that the model excluding small shunts provides a more reliable classification.

Patients with positive bubble study findings who were deemed as cryptogenic (*n* = 83) were discussed in the regional PFO multidisciplinary team (MDT). Following the MDT discussion, 28 patients were deemed candidates for PFO closure. Two declined the procedure. The overwhelming reason for decline was insignificant PFO size. Among the other reasons were MDT decision of non-stroke diagnosis and interval identification of a competing etiology such as vascular stenosis or atrial fibrillation. Out of the 26 patients, 20 had undergone PFO closure by the time of the analysis and the rest were awaiting procedure dates. Table 5 summarizes their demographic data, PFO grade, RoPE scores, and in addition, transesophageal echo findings of high-risk PFO features.

**Table 5:** Characteristics of patients with patent foramen ovale (PFO) closure along with transoesophageal echocardiogram (TOE) high-risk features.

	**Total (*****N =*** **20)**	
	**n**	**%**
**Overall demographics**		
Median age (IQR)	48 (31.8–51.3)	
Sex (male)	13	65.0
Ethnicity (white)	16	80.0
**Risk factors**		
Median BMI (IQR)	27 (24.1–29.8)	
Smoker	6	30.0
Alcohol misuse	0	0.0
History of hypertension	5	25.0
History of diabetes	0	0.0
Hyperlipidaemia	8	40.0
Hypermobile septum	13	65.0
Prominent eustachian tube or Chiari network	7	35.0
Large shunt	20	100.0
PFO tunnel length >10 mm	15	75.0
Two or more high-risk features	20	100.0
**Risk of paradoxical embolism (RoPE) score (>5 pts)**	20	100.0
**Outcome**		
Presence of significant PFO model 1 (≥small)	20	100.0
Presence of significant PFO model 2 (≥moderate)	20	100.0

Although the numbers are too small to test for statistical significance, it is evident that these patients all had high RoPE scores with large PFO and multiple high-risk features in transoesophageal echocardiography (TOE). Further studies with greater sample sizes are warranted to determine whether these findings are replicable and generalizable.

## Discussion

**Figure 4:**
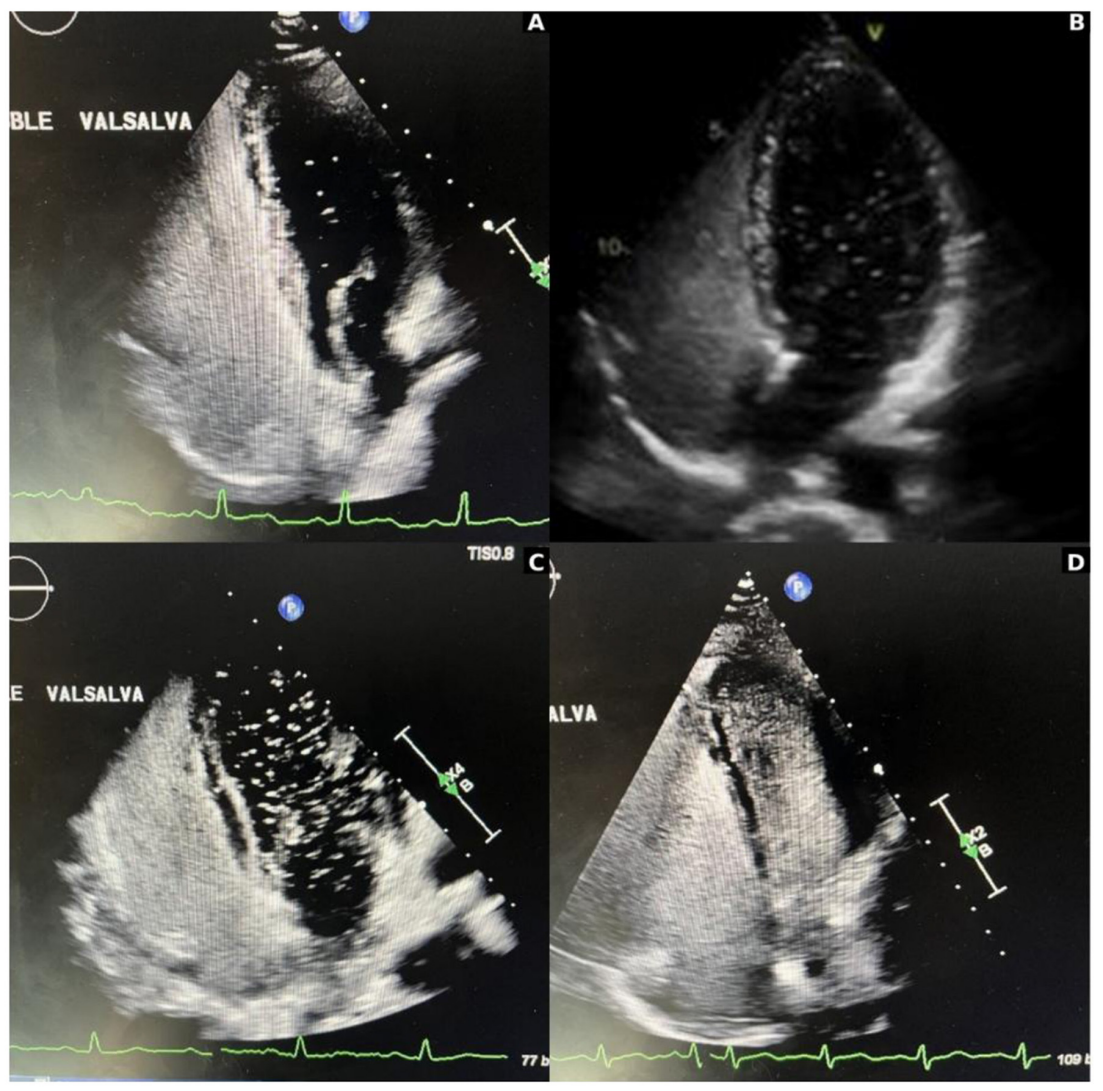
Grading of patent foramen ovale (PFO) on bubble echocardiogram: **(A)** small or grade 1 = 1–5 bubbles; **(B)** moderate or grade 2 = 6–19 bubbles; **(C)** large or grade 3 = ≥20 bubbles; **(D)** very large or grade 4 = near opacification.

PFO is a persistent small flap-like opening between the two cardiac atria. A foramen ovale is a normal interatrial communication present during fetal life. It is bordered by the limbus (embryonic septum secundum) and is guarded by the valve of the fossa ovalis, which is the embryonic septum primum. Typically, the foramen ovale closes after birth as the rising left atrial pressure exceeds right atrial pressure, pushing the valve of the fossa ovalis against the limbus and sealing the connection. However, in 25%−30% of the population, complete anatomical closure does not occur, leaving a persistent channel—known as a PFO—through which blood may shunt from the venous to the arterial system whenever the right atrial pressure surpasses the left. In cases of atrial dilatation, the PFO can stretch, allowing continuous shunting throughout the cardiac cycle (Kheiwa et al., 2020). Bubble echocardiogram is the preferred modality of cardiac imaging to identify and grade the PFO (Figure 4).

Over recent years, PFO has been attributed to approximately 10% of all ischaemic strokes in the 18–60 years age group, and as high as 40% in all cryptogenic stroke cases (Caso et al., 2024; Saver et al., 2017). The proposed mechanism includes paradoxical embolization through right-to-left shunt and thrombus formation within the PFO with subsequent embolization. Landmark trials like RESPECT, REDUCE, and CLOSE have proven that device closure is superior to standard medical therapy. However, subsequent studies have shown that recurrent stroke and TIA rate can range between 2.5% and 3.5%, which is largely attributed to conventional vascular risk factors and newly diagnosed atrial fibrillation (Mariucci et al., 2017). At times, there is real risk of wrongly attributing PFO as the causal factor for the index event where another more conventional etiology can already explain the event. One study showed that almost 40% of patients referred for a bubble echocardiogram had three or more conventional vascular risk factors, and 15% had atrial fibrillation and vascular stenosis combined (Maggiore et al., 2018). This can lead to increased stroke recurrence rate and exposes the patient to undue adverse outcomes related to the procedure.

It is now a well-established practice that a didactic approach is required mainly through an MDT to select patients for device closure. Through evaluation and screening for conventional risk factors, prolonged cardiac rhythm monitoring, and review of imaging, we can ensure appropriate patient selection (Khan et al., 2024). There are two clinical scoring systems, RoPE score and PASCAL classification, which are used in clinical practice to help with decision-making.

The risk of paradoxical embolism (RoPE) study database includes a total of 12 component databases—four enrolled cryptogenic stroke subjects who had PFO, and eight enrolled cryptogenic stroke subjects regardless of PFO status of which the PFO prevalence in these eight studies ranges from 21% to 63%. They pooled data related to patient demographics, cardiovascular risk factors, prior treatment, neuro and vascular imaging, and echocardiographic features of PFO (Thaler et al., 2013). Subsequently, the group used the pooled data from the original study to develop RoPE scores in order to determine PFO attributable fraction correlated to the pathological nature of the PFO related to the index cryptogenic stroke. A high score for a patient with a cryptogenic stroke means a greater likelihood that a PFO is present and probably pathogenic; a lower score makes this less likely or unlikely. It also predicts significantly less stroke recurrence following device closure (Kent et al., 2020).

The PASCAL classification combines the RoPE score with high-risk PFO features, including large right-to-left shunt and atrial septal aneurysm (ASA), to determine the causal relationship between PFO and cryptogenic stroke (Kent et al., 2021).

The SCOPE group developed the PASCAL classification system to evaluate the likelihood of individual benefit from treatment. PASCAL classifies patients with cryptogenic stroke into three categories (Table 6). The study showed hazard ratios (HRs) and absolute risk reduction (ARR) for the categories unlikely, possible, and probable following device closure. The HR and ARR in the “unlikely” group were 1.14 and −0.7%, respectively, at 2 years following device closure. For the “probable” group, the values were 0.10 and 2.1%, respectively. The study also showed that patients with a combination of large right-to-left shunt showed the greatest ARR of 5.5%. Both the American Stroke Association and European Stroke Organization guidelines put high emphasis on “high risk anatomical and physiological features” in patient selection for PFO closure (American Stroke Association, 2021; Caso et al., 2024; Kent et al., 2021).

**Table 6:** PASCAL classification.

**High RoPE score (≥7)**	**High-risk PFO feature (LS and/or ASA)**	**PFO-related stroke**
Absent	Absent	Unlikely
Absent	Present	Possible
Present	Absent	Possible
Present	Present	Probable

RoPE, risk of paradoxical embolization; PFO, patent foramen ovale; LS, large shunt; ASA, atrial septal aneurysm.

Nakayama et al. (2019) highlighted high-risk anatomical and physiological features of PFO that are associated with cryptogenic stroke. They also proposed a simple scoring system where a score of ≥2 signifies association of PFO with cryptogenic stroke with 91% sensitivity and 80% specificity (Table 7) (Nakayama et al., 2019).

**Table 7:** High-risk patent foramen ovale (PFO) score calculator.

**High risk PFO features**	**Score**
Long-tunnel PFO 10 mm or more	1
Hypermobile interatrial septum (a floppy septum with excursion ≥ 5 mm in every heartbeat)	1
Eustachian valve or Chiari's network	1
Large right-to-left shunt during Valsalva maneuver (20 or more bubbles in left atrium with in first three cardiac cycles)	1
Low-angle PFO ≤ 10^0^	1

**Figure 5:**
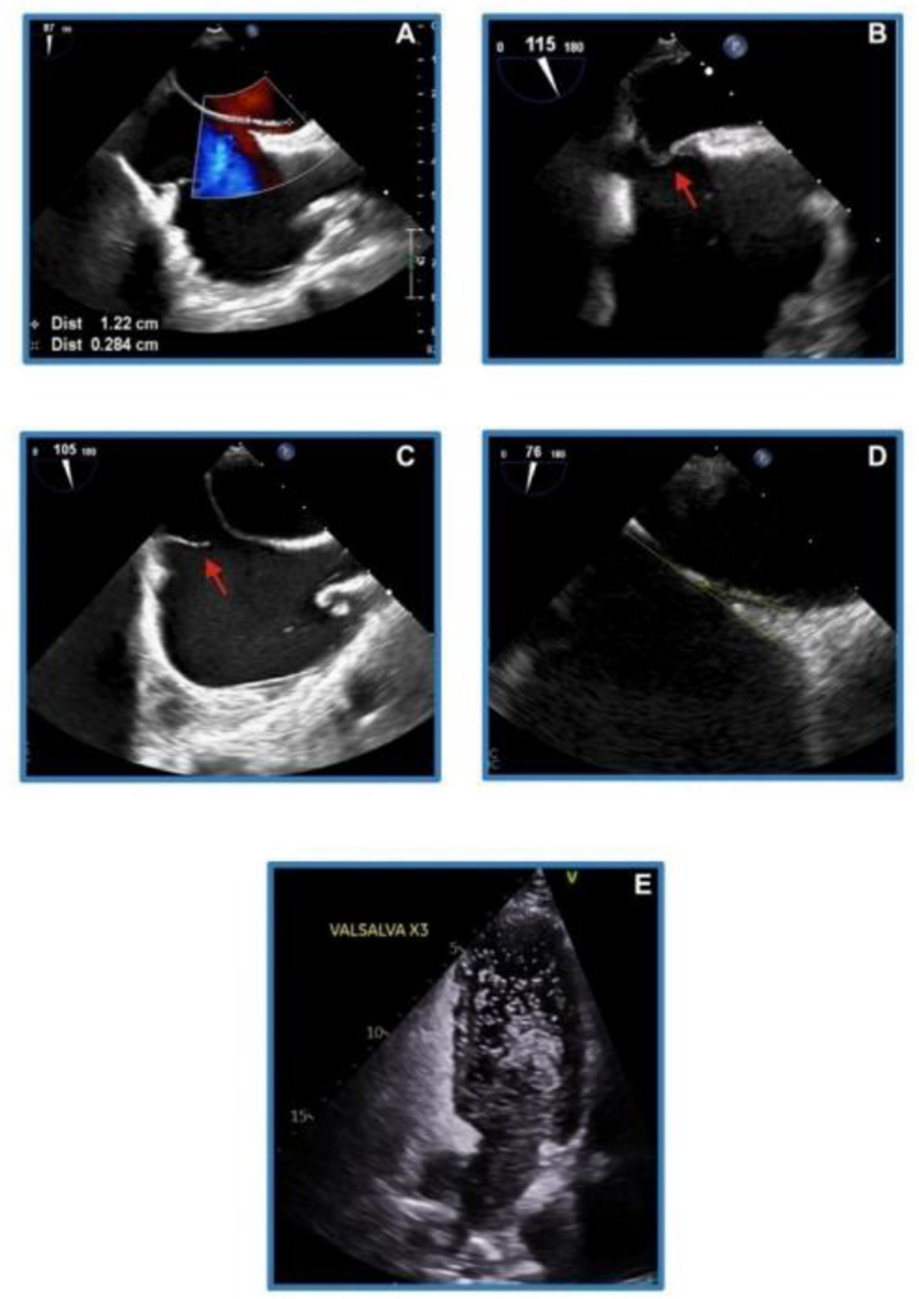
High-risk PFO anatomical features. **(A)** Measurement of the PFO showing a height (maximum separation between septum primum and septum secundum) >2 mm, and a tunnel length (maximum overlap) >10 mm. **(B)** Aneurysmal interatrial septum, defined as septal excursion ≥10 mm during the cardiac cycle. **(C)** Prominent eustachian valve, defined as a linear structure protruding ≥10 mm into the right atrium. **(D)** Low-angle PFO configuration, characterized by a sharp angle (< 10°) between the inferior vena cava (IVC) inflow and the PFO tunnel, assessed in an imaging plane containing both the IVC and interatrial septum. **(E)** Large right-to-left shunt on bubble transthoracic echocardiogram, defined as >20 microbubbles appearing in the left atrium within three cardiac cycles of right atrial opacification. *Note* **(A–D)** Transoesophageal echocardiogram (TOE) images; **(E)** transthoracic echocardiogram. RA, right atrium; LA, left atrium; RV, right ventricle; LV, left ventricle.

Other high-risk anatomical features include a 10 mm septal excursion from the midline into the right or left or 15 mm total excursion between the right and left atrium and PFO height of ≥2 mm (see Figure 5).

Trials including higher-risk PFO patients and with a longer follow-up [CLOSE trial (9), DEFENSE-PFO trial (10), REDUCE trial (11), RESPECT study (5)] demonstrated a significant reduction in ischaemic stroke recurrence with PFO closure as compared with antithrombotic treatment (antiplatelet or anticoagulant therapy) (Radico et al., 2021). A recent meta-analysis reported an annual stroke incidence of 0.48 per 100 person-years following PFO closure, compared to 1.26 per 100 person-years with medical therapy alone. The estimated number needed to treat over five years to prevent one stroke was 24 in the general (unselected) population and 13 among patients with high-risk PFO. Greater stroke risk reduction was observed in patients under 45 years of age, males, and those with large shunts, although there was no significant interaction between subgroups (Giacoppo et al., 2018). There is no denying that patients with high-risk PFO features benefit most from device closure. However, the majority of these high-risk features are only identifiable through TOE, which has logistic and cost implications. Therefore, it is important that we select our patients well prior to TOE. Currently there is no clinical scoring system or pathway that clinicians can use as a tool to predict high-risk PFO features. The original RoPE database collected data on echocardiographic variables; however, they were not included in the RoPE score.

Our study showed that patients with an identified stroke cause had a higher burden of traditional cardiovascular risk factors and more frequent lacunar strokes or TIAs, suggesting a clear vascular etiology. In contrast, cryptogenic stroke patients more often had cortical strokes and elevated RoPE scores (≥6, *p*-value < 0.0001), supporting a possible embolic mechanism. Despite this, the prevalence of PFO did not differ between groups, indicating that the causal relationship between cryptogenic stroke and PFO cannot be established by PFO presence alone and depends on additional clinical and anatomical factors. In our study, smoking (*p*-value 0.03), HTN (*p*-value 0.03) and alcohol (*p*-value 0.02) misuse was strongly associated with hemodynamically insignificant PFO, supporting the notion that in stroke patients with established cardiovascular risk factors, PFO is less likely to be causative. This underscores the importance of comprehensive risk assessment when evaluating the clinical relevance of PFO in stroke etiology.

We also explored whether the converse is true where a low RoPE score can predict non-cryptogenic stroke or a known etiology. Our data showed that current smoking, hypertension, hyperlipidaemia, and non-stenotic atherosclerosis were significantly associated with a stroke with etiology. Importantly, a RoPE score ≤ 5 remained independently associated with a known etiology in multivariable analysis, even after adjusting for relevant covariates (aOR: 3.91, *p* < 0.01). The final model demonstrated moderate discriminatory ability (AUC = 0.742), supporting the utility of the RoPE score in distinguishing between cryptogenic and non-cryptogenic stroke mechanisms.

We explored the correlation between RoPE score and PFO grade on bubble echocardiogram. Our study showed higher RoPE scores (≥6) and the presence of cortical stroke were significantly associated with the presence of a PFO with a grade ≥ Small. RoPE score ≥6 had an OR of 5.33 (95% CI: 1.56–18.16, *p* < 0.01) and cortical stroke had an OR of 4.89 (95% CI: 1.75–13.65, *p* < 0.01). These associations were even stronger when moderate-or-greater shunts were considered. RoPE score ≥6 had an OR of 15.06 (95% CI: 1.89–120.20, *p* = 0.01), and cortical stroke had an OR of 13.20 (95% CI: 2.85–61.24, *p* < 0.01). In multivariable models RoPE score remained an independent predictor of significant PFO (an OR: 5.39 for small-or-greater shunt, *p* < 0.01; an OR: 15.95 for moderate-or-greater shunt, *p* < 0.01). Model discrimination improved when excluding small shunts (AUC = 0.725 vs. 0.666), suggesting that moderate or larger shunts are more reliably associated with embolic stroke features. These findings support the value of the RoPE score and cortical stroke phenotype in identifying clinically relevant higher-grade PFOs.

Of the cryptogenic stroke patients with a positive bubble study (*n* = 83), 28 were selected for PFO closure following MDT review and two declined the procedure. The majority of exclusions were due to insignificant PFO size or identification of alternative etiologies. Of the 26 patients selected, 20 had undergone the procedure at the time of analysis. All had high RoPE scores (≥6), large right-to-left shunts (20 or more bubbles), and at least two other high-risk TOE features—most commonly PFO tunnel length >10 mm (75%), hypermobile septum (65%), and prominent eustachian valve or Chiari network (35%). Although the sample size precluded statistical testing, these findings suggest that MDT decisions for closure strongly aligned with current criteria emphasizing embolic phenotype, high RoPE scores, and anatomical and physiological high-risk features. Larger prospective studies or subgroup analysis of existing studies are needed to assess the reproducibility and broader applicability of RoPE scores for the prediction of high-risk PFO.

This study has several limitations that should be considered when interpreting the findings. This is a retrospective single-center study design that limits causal inference and introduces the potential for selection and information biases. Although rigorous chart review and inclusion criteria were applied, misclassification of cryptogenic stroke due to incomplete workup or delayed diagnostic yield (e.g., paroxysmal atrial fibrillation) remains a possibility. The sample size was modest, limiting the statistical power to detect more granular associations and precluding robust subgroup analysis of high-risk PFO anatomical features. External generalizability may be limited given the relatively homogeneous population from a regional stroke unit in the United Kingdom. The demographic and risk factor profile may not reflect broader, more diverse populations where PFO prevalence and stroke etiologies may differ. Prospective, multicenter studies with larger sample sizes are needed to validate and expand upon these findings.

## Conclusion

Correlation between higher RoPE score and high-risk anatomical and physiological features of PFO is a novel approach. Our study shows the potential for RoPE score to be utilized beyond its original intended scope. Further dedicated studies correlating various RoPE score thresholds with high-risk TOE findings is required to validate findings from this study and establish whether the RoPE score has extended utility in patient selection in cryptogenic stroke and PFO.

## Data Availability

The raw data supporting the conclusions of this article will be made available by the authors, without undue reservation.

## References

[B1] Adams H. P. Bendixen B. H. Kappelle L. J. Biller J. Love B. B. Gordon D. L. . (1993). Classification of subtype of acute ischemic stroke. Definitions for use in a multicenter clinical trial. TOAST. Trial of Org 10172 in Acute Stroke Treatment. Stroke. 24, 35–41. doi: 10.1161/01.STR.24.1.35 7678184

[B2] Akagi T. (2021). Transcatheter closure of patent foramen ovale: Current evidence and future perspectives. J Cardiol. 77, 3–9. doi: 10.1016/j.jjcc.2020.09.005 33144025

[B3] American Stroke Association (2021). Understanding Diagnosis and Treatment of Cryptogenic Stroke an Updated Health Care Professional Guide. Dallas (TX): American Stroke Association.

[B4] Arsava E. M. Ballabio E. Benner T. Cole J. W. Delgado-Martinez M. P. Dichgans M. . (2010). The Causative Classification of Stroke system. Neurology. 75, 1277–84. doi: 10.1212/WNL.0b013e3181f612ce 20921513 PMC3013495

[B5] Caso V. Turc G. Abdul-Rahim A. Castro P. Hussain S. Lal A. . (2024). European Stroke Organisation (ESO) Guidelines on the diagnosis and management of patent foramen ovale (PFO) after stroke. Eur Stroke J. 9:800–34. doi: 10.1177/23969873241247978 38752755 PMC11569559

[B6] Diener H. C. Sacco R. L. Easton J. D. Granger C. B. Bernstein R. A. Uchiyama S. . (2019). Dabigatran for Prevention of Stroke after Embolic Stroke of Undetermined Source. New England Journal of Medicine. 380, 1906–17. doi: 10.1056/NEJMoa1813959 31091372

[B7] Giacoppo D. Caronna N. Frangieh Antonio H. Michel J. Andò G. Tarantini G. . (2018). Long-term effectiveness and safety of transcatheter closure of patent foramen ovale compared with antithrombotic therapy alone: a meta-analysis of six randomised clinical trials and 3,560 patients with reconstructed time-to-event data. EuroIntervention. 14:857–67. doi: 10.4244/EIJ-D-18-00341 29901447

[B8] Hart R. G. Sharma M. Mundl H. Kasner S. E. Bangdiwala S. I. Berkowitz S. D. . (2018). Rivaroxaban for Stroke Prevention after Embolic Stroke of Undetermined Source. New England Journal of Medicine. 378, 2191–201. doi: 10.1056/NEJMoa1802686 29766772

[B9] Kent D. M. Ruthazer R. Weimar C. Mas J. L. Serena J. Homma S. . (2013). An index to identify stroke-related vs. incidental patent foramen ovale in cryptogenic stroke. Neurology. 81, 619–25. doi: 10.1212/WNL.0b013e3182a08d59 23864310 PMC3775694

[B10] Kent D. M. Saver J. L. Kasner S. E. Nelson J. Carroll J. D. Chatellier G. . (2021). Heterogeneity of Treatment Effects in an Analysis of Pooled Individual Patient Data From Randomized Trials of Device Closure of Patent Foramen Ovale After Stroke. JAMA. 326, 2277. doi: 10.1001/jama.2021.20956 34905030 PMC8672231

[B11] Kent D. M. Saver J. L. Ruthazer R. Furlan A. J. Reisman M. Carroll J. D. . (2020). Risk of Paradoxical Embolism (RoPE)–Estimated Attributable Fraction Correlates With the Benefit of Patent Foramen Ovale Closure. Stroke. 51, 3119–23. doi: 10.1161/STROKEAHA.120.029350 32921262 PMC7831886

[B12] Khan M. Miller M. Mccarthy P. Tsai Jenny P. Merhi W. Berkompas D . (2024). Multidisciplinary Approach to Patent Foramen Ovale Closure for Cryptogenic Stroke. Neurol Clin Pract. 14(4). doi: 10.1212/CPJ.0000000000200319 PMC11141343 38826798

[B13] Kheiwa A. Hari P. Madabhushi P. Varadarajan P. (2020). Patent foramen ovale and atrial septal defect. Echocardiography. 37:2172–84. doi: 10.1111/echo.14646 33368546

[B14] Maggiore P. Bellinge J. Chieng D. White D. (2018). Lan Nick SR Jaltotage B . Ischaemic Stroke and the Echocardiographic “Bubble Study”: Are We Screening the Right Patients? Heart Lung Circ. 2019; 28(8):1183–9. doi: 10.1016/j.hlc.2018.07.007 30131285

[B15] Mariucci E. Donti A. Salomone L. Marcia M. Guidarini M. Formigari R. . (2017). Recurrent Stroke after Transcatheter PFO Closure in Cryptogenic Stroke or Tia: Long-Term Follow-Up. Cardiol Res Pract. 2017, 1–10. doi: 10.1155/2017/9849425 29430320 PMC5753007

[B16] Nakayama R. Takaya Y. Akagi T. Watanabe N. Ikeda M. Nakagawa K. . (2019). Identification of High-Risk Patent Foramen Ovale Associated With Cryptogenic Stroke: Development of a Scoring System. Journal of the American Society of Echocardiography. 2019; 32(7):811–6. Available from: https://libkey.io/10.1016/j.echo.03, 021. doi: 10.1016/j.echo.2019.03.021 31130417

[B17] Park S. Oh J. K. Song J. Kwon B. Kim B. J. Kim J. S. . (2021). Transcranial Doppler as a Screening Tool for High-Risk Patent Foramen Ovale in Cryptogenic Stroke. Journal of Neuroimaging. 31, 165–70. doi: 10.1111/jon.12783 32896963

[B18] Radico F. Foglietta M. Di Fulvio M. Appignani M. Rossi S. Angelis M. . (2021). The ‘dreaded PFO': anatomical and functional features of high risk for stroke. European Heart Journal Supplements. 2021; 23:E189–93. doi: 10.1093/eurheartj/suab119 35233215 PMC8876301

[B19] Sakai Y. Lehman V. T. Eisenmenger L. B. Obusez E. C. Kharal G. A. Xiao J. . (2022). Vessel wall MR imaging of aortic arch, cervical carotid and intracranial arteries in patients with embolic stroke of undetermined source: A narrative review. Front Neurol. 13. doi: 10.3389/fneur.2022.968390 PMC9366886 35968273

[B20] Saver J. L. Carroll J. D. Thaler D. E. Smalling R. W. MacDonald L. A. Marks D. S. . (2017). Long-Term Outcomes of Patent Foramen Ovale Closure or Medical Therapy after Stroke. New England Journal of Medicine. 377, 1022–32. doi: 10.1056/NEJMoa1610057 28902590

[B21] Strambo D. Sirimarco G. Nannoni S. Perlepe K. Ntaios G. Vemmos K. . (2021). Embolic Stroke of Undetermined Source and Patent Foramen Ovale. Stroke. 52, 1643–52. doi: 10.1161/STROKEAHA.120.032453 33784832

[B22] Tao L. Li X. Q. Hou X. W. Yang B. Q. Xia C. Ntaios G. . (2021). Intracranial Atherosclerotic Plaque as a Potential Cause of Embolic Stroke of Undetermined Source. J Am Coll Cardiol. 77, 680–91. doi: 10.1016/j.jacc.2020.12.015 33573737

[B23] Thaler D. E. Di Angelantonio E. Di Tullio M. R. Donovan J. S. Griffith J. Homma S. . (2013). The Risk of Paradoxical Embolism (RoPE) Study: Initial Description of the Completed Database. International Journal of Stroke. 8, 612–9. doi: 10.1111/j.1747-4949.2012.00843.x 22883936 PMC4060865

[B24] World Stroke Organization (2025). World Stroke Day 2025: Impact of Stroke. [cited 2025 Jun 19]; Available from: https://www.world-stroke.org/world-stroke-day-campaign/about-stroke/impact-of-stroke#:~:text=World%20Stroke%20Organization,treatment%20and%20rehabilitation%20of%20stroke.

